# Psychological Profiles in the Prediction of Leukocyte Telomere Length in Healthy Individuals

**DOI:** 10.1371/journal.pone.0165482

**Published:** 2016-10-27

**Authors:** Louisia Starnino, Lambert Busque, Jean-Claude Tardif, Bianca D’Antono

**Affiliations:** 1 Research Center, Montreal Heart Institute, Montreal, Canada; 2 Department of Psychology, Université du Québec à Montréal, Montreal, Canada; 3 Research Center, Hematology Division, Hôpital Maisonneuve-Rosemont, Université de Montréal, Montreal, Canada; 4 Department of Medicine, Montreal Heart Institute and Université de Montréal, Montreal, Canada; 5 Department of Psychology, Université de Montréal, Montreal, Canada; University of Newcastle, UNITED KINGDOM

## Abstract

**Background:**

Shorter telomere length (TL) may signal premature cellular aging and increased risk for disease. While depression and psychosocial stress have been associated with shorter telomeres, other psychological risk factors for cardiovascular disease have received less attention.

**Purpose:**

To evaluate the association between TL and psychological risk factors (symptoms of anxiety and depression, hostility and defensiveness traits) for heart disease, and to examine whether chronological age and sex moderate the associations observed.

**Methods:**

132 healthy men and women (M_age_ = 45.34 years) completed the Marlowe-Crowne Social Desirability Scale, the Beck Depression Inventory II, The Beck Anxiety Inventory and the Cook-Medley Hostility Scale. Relative TL was measured by quantitative polymerase chain reaction (PCR) of total genomic DNA samples. A series of hierarchical linear regressions were performed controlling for pertinent covariates.

**Results:**

Shorter TL was observed among individuals high in defensiveness (β = -.221) and depressive symptoms (β = -.213), as well as in those with less hostility (β =.256) and anxiety (β =.220)(all Ps<.05). Psychological variables explained 19% of the variance over and above that explained by covariates (age, sex, exercise, alcohol consumption, systemic inflammation, and 24-hr mean arterial pressure). Age moderated the relation between TL and defensiveness (β =.179, p =.03). Sex did not influence any of the relations.

**Conclusions:**

Telomere length is associated with psychological burden though the direction of effect differs depending on the psychological variables under study. Further research is needed to determine the reasons for and implications of these seemingly contradictory findings.

## Introduction

Psychological distress and personality traits, such as depression and hostility have been shown to increase the risk of developing and dying from cardiovascular disease (CVD) or related disorders [1–3]. The mechanisms underlying this increased risk remain for the most part speculative. While individuals possessing these various psychological traits frequently exhibit poorer health habits [4, 5], their association with CVD are typically independent of lifestyle factors [6–8]. Psychological variables may impact cardiovascular health through their influence on multiple bodily functions [9, 10].

Among the emerging biological risk factors for CVD is shortened telomere length (TL)[11, 12]. Telomeres are specialized DNA-protein repetitive sequences (TTAGGG) that cap eukaryotic chromosome ends during mitotic cell proliferation in order to prevent end-to-end recombination, deterioration, or fusion with neighbouring chromosomes, and as such, play an integral role in preventing the loss of genetic data [13]. With advancing age (and multiple replication cycles), telomeres tend to shorten. Extensive shortening of telomeres may lead to cellular damage or senescence and increased risk of disease [14]. Telomere length (TL) has thus been suggested as a marker of cellular aging [15] with shorter TL being associated with unfavorable age-related diseases such as an increased rates of CVD and cancer [16–19].

While not extensively studied, there is increasing evidence that psychological distress may contribute to premature cellular aging, as measured by shortened TL [20–22]. Mood disorders, and in particular depression, are among the most widely studied psychological risk factors with respect to TL. Several studies have observed shorter TL among individuals with major depression (MDD) and/or recurrent depressive episodes [22–26] as well as in individuals with bipolar disorder [27] in comparison with healthy individuals without diagnosed psychological disorders. For example, Verhoeven and colleagues [28] reported significantly shorter TL among remitted and current MDD patients compared to healthy controls. Severity and duration of current depressive symptoms were similarly associated with shorter TL. However, several studies that have employed self-report questionnaires of depression have not observed such relations [29–32].

Fewer studies have examined TL as a function of anxiety, and obtained mixed results [20, 21, 24, 25, 33]. For example, young women (but not men) diagnosed with general anxiety disorder (GAD) and panic disorder (PD) were shown to exhibit shorter TL than individuals without anxious affect [24]. Similarly, in a large study of 42–69 year old women enrolled in the Nurse’s Health study, high self-reported phobic anxiety was found to be associated with significantly shorter TL after controlling for paternal age-at-birth, smoking, BMI and physical activity [20]. On the other hand, no significant association was found between self-reported anxiety symptoms (Beck Anxiety Inventory (BAI)) and TL among 91 individuals also suffering from depression [25].

Only one study to date has investigated hostility and TL. In a healthy sample of middle-aged and older adults (n = 543; 53–76 years of age), Brydon and colleagues [34] reported that high cynical hostility was associated with shorter TL in men but not women.

While investigations to date are interesting, they have typically been performed in clinical samples. However, the evaluation of healthy individuals may provide insights into the pathophysiological pathways contributing to disease states unconfounded by the reciprocal effects of disease processes on psychological health. Moreover, while mood disorders and TL have been the focus of multiple studies, personality traits have all but been ignored, despite their importance to CVD risk and prognosis. Starkweather and colleagues [35] have argued that it is important to consider socio-demographic and psychosocial factors that may moderate, mediate, or otherwise confound results in studies of TL attrition. Yet, the independence of associations of TL with one psychological risk factor from other psychological variables is unknown. Finally, sex and age differences exist in TL, psychological risk factors and CVD risk and may influence the associations observed among these variables. Aging is associated with shorter telomeres [36] and differential prevalence rates of psychological distress [37]. Women typically have longer TL than men [38], though this may vary according to chronological age [39, 40]. They also show a greater prevalence of depression [41] and anxiety [42]. Sex differences in certain personality traits have also been reported [43]. The influence of sex and/or age on the relation between psychological factors and TL has not been frequently examined. One study of 974 individuals with and without a diagnosis of anxiety disorder aged 30–87 found no significant differences in TL among anxious participants versus controls in the overall sample, but did show shorter TL compared to controls among older patients (≥ 48 years) in subgroup analyses. The authors suggested that the stress related to having an anxiety disorder may need to cumulate over time for accelerated telomere shortening to occur [44]. As mentioned earlier, sex differences in the relation between TL and hostility [34] and anxiety [24] have also been found, albeit in opposite directions.

The objectives of this study are thus to evaluate the independent associations of psychological risk factors (symptoms of anxiety and depression, and traits of hostility and defensiveness) with TL in 132 healthy participants, and to examine whether these relations differ as a function of sex and/or age. Defensiveness, measured most often using the Marlowe Crowne Social Desirability Scale, refers to a personality trait characterized by the avoidance, denial or repression of information (e.g., negative affects, physical symptoms, poor performance) perceived as threatening to the individual. Individuals with this trait are more prone to adopt socially desirable behaviors such as conforming to the opinion of others or lying about inappropriate behaviors and suboptimal performance in order to secure social bonds and/or to protect a vulnerable self-esteem [45, 46]. It has been shown to increase risk for CVD morbidity [47, 48], mortality [49] and hypertension [6, 50–52]. Based on existing literature, it is expected that TL will be shorter in individuals with higher vs. lower levels of psychological distress independently of conventional behavioural and biological risk factors. While differences are expected as a function of sex and/or age, the paucity and conflicting nature of data to date precludes firm hypotheses about the direction of effects.

## Methods

Current analyses are part of a larger prospective study examining associations of psychological and psychophysiological factors with intermediary risk factors for CVD. For work published from this study, see [53–60]. The results presented are based on data obtained from the follow-up evaluation (time 2) of the prospective study.

### Participants

#### Time 1

One hundred and ninety nine healthy adult men (n = 81) and women (n = 118) were recruited from advertisements in community centers and newspapers within the greater Montreal area. Eligibility criteria included: (a) no utilization of mental health services within the past year, (b) no current or known health problems or use of medication known to affect cardiovascular, immune, or neuroendocrine functions, (c) no learning or cognitive disabilities capable of impairing the ability to complete questionnaires or understand instructions and (d) not currently on hormone replacement therapy. To guarantee a broad age distribution, participants were selected to provide approximately three equal age groups (18–34 years; 35-44 years; 45–65 years). Women were over-sampled to include a sufficient number of post-menopausal women for a separate component of the study not examined here.

#### Time 2

Three years later (1031 ± 107.5 days), 141 of the original 199 participants were evaluated. Twenty participants from the original sample could not be reached. Six were not eligible to participate for medical reasons including pregnancy (n = 3), cancer (n = 2), and sleep apnea (n = 1), while 35 declined due to lack of interest (n = 16), scheduling issues (n = 15), or perception that the protocol was too demanding (n = 4). Missing data for TL led to a final sample of 132 participants (54 men, 78 women; M_age_ = 45 ±11.6 yrs).

#### Procedure (Session 2)

Participants were given an appointment at the Montreal Heart Institute. To control for circadian rhythms in physiological activity, all visits began at 8:00 A.M. on a weekday. Participants were asked to abstain from eating and drinking (other than water) and refrain from any strenuous physical activity and smoking for 12h prior to their appointment, as well as abstain from alcohol or other drug intake for 24h. Participants who did not adhere to these instructions on the day of testing or who presented physical symptoms (such as cough) on the day of testing were sent home and a new appointment was scheduled.

In the laboratory, participants were tested by a same-sex research assistant. Once written informed consent was obtained, waist circumference, height and weight were measured. Electrodes for electrocardiogram (ECG) monitoring were then attached in a bipolar configuration to the lower side of the rib cage, and a ground electrode was placed on the left hip. A blood pressure cuff was placed on the non-dominant arm. Questionnaires were administrated to obtain information on sociodemographic, medical, and psychological profile. A 10-min baseline period ensued, during which subjects rested quietly, followed by a blood draw. Participants were then exposed to a laboratory stress protocol that included four 5 minute interpersonal stressors, each followed by 5-minute post-stress (recovery) periods (for details of the stress protocol see [55]). Following the laboratory visit, ambulatory BP and ECG were obtained during a 24-h period. Participants received 250$ at time 2 covering expenses for time and travel. The Research and Ethics Board of the Montreal Heart Institute approved this study.

### Questionnaires

#### Socio-demographic variables

Data on sex, age, body mass index (BMI), marital status, annual income and years of schooling was obtained at the beginning of experimentation.

#### Health information

The questionnaires assessed behavioural risk factors relating to tobacco caffeine and alcohol consumption as well as physical activity.

#### Measurement of psychological variables

Beck Anxiety Inventory (BAI; [61]): a 21-item self-report questionnaire that instructs participants to rate symptoms of anxiety experienced over the past two weeks using a 4-point scale ranging from “Not at all”, to “Severely”. Scores greater than or equal to 8 indicate mild to severe anxiety. The measure has excellent internal consistency (α = 0.92) and one week test–retest reliability (r = 0.75) [61]. In the current sample, internal consistency was α =.88; and 3-year test-retest reliability was 0.72.

Beck Depression Inventory-II (BDI-II; [62]): a 21-item self-report questionnaire that instructs participants to rate symptoms of depression experienced over the past two weeks, using a 4-point scale ranging from “Not at all” to “Severely”. Scores greater than 13 indicate mild to severe symptoms of depression. The measure has excellent internal consistency (α = 0.91) and a one-week test–retest reliability (r = 0.93) [63]. In the current sample, internal consistency was α =.89, and 3-year test-retest reliability was 0.73

The Cook–Medley Hostility Inventory (CMHo; [64]): a 50-item empirically derived self-report scale that measures a cynical, mistrustful attitude towards others. It is answered using a true–false format. The internal consistency of this instrument (α = 0.82–0.86) [65], and test-retest reliability (rs>0.85) [66] are excellent. In the current sample it had an internal consistency of α = 0.83 and 3-year test-retest reliability was 0.84.

The Marlowe-Crowne Social Desirability scale (MCSD; [67]): is a 33 true or false item scale that assesses the tendency to respond in a culturally sanctioned and desirable manner. The items present behaviors that are desirable but infrequent (e.g., ‘‘I am always courteous, even to people who are disagreeable”) and behaviors that are undesirable but frequent (e.g., ‘‘I like to gossip at times”). The MCSD has been found to have excellent internal consistency (0.88) and one month test–retest reliability (0.86) [68]. In the current sample, internal consistency was α = 0.75, and 3-year test-retest reliability was 0.85.

#### Physiological variables

Telomere length. DNA was extracted from peripheral blood leukocytes using standard methods, and TL measured as described by Cawthon [69] with minor modifications [70]. Briefly, using two primer pairs that target telomeric hexamer repeats and a single copy gene (*36B4*, which encodes acidic ribosomal phosphoprotein PO), the telomere repeat copy number (T) to single copy gene copy number (S) ratio was calculated for each DNA sample. The T/S ratio of each experimental DNA sample was obtained relative to a reference DNA sample (obtained from a single individual and used for standard curve generation) whose T/S ratio is 1. For each sample DNA, T and S Sybr^®^Green PCR reactions were run on an ABI SDS 7000 real-time PCR apparatus (Applied Biosystems, CA, USA). Each 25μL PCR reaction contained the following: 25ng DNA, 12.5μL of Sybr^®^Green PCR master mix (ABI), and 450nM of each primer for Treactions(Tel1b:CGGTTTGTTTGGGTTTGGGTTTGGGTTTGGGTTTGGGTT;Tel2b:GGCTTGCCTTACCCTTACCCTTACCCTTACCCTTACCCT) or 300nM of forward primer 36B4u (CAGCAAGTGGGAAGGTGTAATCC) and 500nM of reverse primer 36B4d (CCCATTCTATCATCAACGGGTACAA) for S amplifications. Serial dilutions (6.48 to 83.3ng/μL) of the reference DNA were used to generate standard curves to calculate the amount of both T and S for each sample DNA. The relative T/S ratio of each sample was obtained by dividing the amount of T by the amount of S. All samples were measured in triplicate, and their mean was used for analyses.

Various other physiological variables were examined as potential confounders in primary or post-hoc analyses.

Inflammatory markers. C-reactive protein (CRP) was measured from serum using the Siemens (formerly Dade Behring) CardioPhase hsCRP assay (Siemens Healthcare Diagnostics Products GmbH, Marburd, Germany) on the BN ProSpec Nephelometer (Siemens Healthcare Diagnostics Products GmbH). The minimal detectable hsCRP concentration was 0.18 mg/L.

Metabolic Syndrome (MS). Participants with metabolic syndrome (MS) were identified according to the National Cholesterol Education Program-Third Adult Treatment Panel (NCEP-ATP-III, 2002) criteria for BP, waist circumference, glucose, triglyceride, and high density lipoprotein levels (HDL). Serum samples were analyzed for lipids and glucose at the MHI. These determinations were made using respective reagent Flex on the multianalyzer Dimension RxL Max (Dade Behring Diagnostics, Marburg, Germany) with heparinized plasma, as simultaneously as possible following blood draw. Waist circumference was obtained using a standard measuring tape. Ambulatory BP measures were obtained every 20 min in the daytime and every hour from 22h00 to 6h00, using Spacelab Ambulatory Blood Pressure Units. The BP measures were based on values averaged over 24 h.

Stress reactivity measures. Systolic (SDP) and diastolic blood pressure (DBP) were measured in the laboratory using the AccutorPlus automated blood pressure monitor from Datascope (Datascope Inc., Montvale, NJ) using a standard inflatable cuff placed on the participant’s nondominant arm. The laboratory-based baseline BP was based on the average of two readings obtained during the last 5 min of a 10-min rest period prior to preparations for venipuncture. BP, heart rate (HR) and heart-rate variability (HRV); high frequency heart rate variability (HF-HRV), normalized high frequency (HFnu), low frequency (LF-HRV), and ratio (LF/HF)) readings were averaged over each baseline, stress, and post-stress period. The four stress periods were averaged to create a composite stress and post-stress score, as per our prior research (see for more details see [55, 59]. Stress reactivity (stress−baseline) and recovery (post-stress score−baseline) change scores were then created as per established methods [71]. Finally, in order to minimize the impact of individual differences in baseline values on the change scores, the latter were further regressed on baseline values.

Salivary cortisol was obtained throughout the protocol using Salivettes (Sarstedt, Montreal, Canada) containing a piece of absorbent gauze. Participants chewed on the swab for 45 seconds until it was saturated with saliva. Samples were transferred to a -20°C freezer and analyzed off-site using an enzyme immunoassay kit (Assay Designs, Inc., Ann Arbor, MI) in duplicates (see Juster et al., 2013, [57] for more detail).

### Analyses

In order to increase normality, scores for BDI-II, BAI, and CRP underwent log transformations. Pearson correlations were used to examine the relations between TL and potential covariates (for example, smoking, exercise, alcohol consumption, marital status, annual income, CRP, BMI, mean arterial BP (MAP)) as determined from previous literature. Regular physical activity, for example, has been associated with significant health benefits [72] including longer TL [73]. Alcohol consumption, for its part, has been shown to correlate negatively with TL [74, 75]. Since telomeres are believed to be highly sensitive to damage by oxidative stress [76] and CRP increases generation of oxygen free radicals by neutrophils [77] it is biologically plausible that CRP could damage telomeric DNA and contribute to the shortening of TL. Indeed, CRP levels have been shown to be negatively correlated with TL [78, 79]. High blood pressure is a well-known risk factor for heart disease, and is associated with hostility [80], defensiveness [81], anxiety [82], depression [83] and shortened TL [84]. Given the limited sample size, only variables correlating with TL at p≤.200 were retained as covariates. These included exercise, number of alcoholic beverages/week, CRP, and 24-hour MAP.

Pearson correlations were used to examine univariate relations between TL and each psychological variable.

The multivariate associations of TL with the psychological factors were examined using hierarchical linear regressions. In Block 1, age, sex, and the selected covariates were entered. Psychological variables were entered simultaneously in Block 2 to examine their independent associations with TL.

To examine whether age and/or sex moderated the relations between psychological variables and TL, four separate hierarchical regressions were performed. Sex, age and other covariates were entered in Block 1, while the psychological variable was entered individually in Block 2. The 2-way interaction terms of sex or age with the psychological variable were entered stepwise in Block 3. Interaction terms were created from centered data. Statistical significance was at p<0.05. When there were significant interaction effects, simple slopes analyses were performed with lower and higher estimates for age and psychological profile based on values ±1 SD from the mean [85].

## Results

### Sample characteristics

Table 1 presents mean values for the demographic and behavioural sample characteristics

**Table 1:** Characteristics of participants.

Participants	(n = 132)
Age (years ± SD)	45.34 (11.16)
Glasses of alcohol/week (±SD)	3.84 (5.38)
Smoker n (%)	19 (14%)
**Marital Status n (%)**	
Single	55 (42%)
Married/living with someone	56 (42%)
Separated/divorced/widowed	21 (16%)
**Annual family income n (%)**	
≤ $29,999	41 (31%)
$30 000–59 999	45 (34%)
≥ $60 000	46 (35%)
***Physiological measures***	
Metabolic Syndrome *n (%)	16 (12%)
Body Mass Index (kg/m^2^ ±SD)	25.35 (5.00)
Mean arterial pressure 24 hours (mmHg ± SD)	85 (7.22)
CRP (mg/L ±SD)	1.76 (2.66)
TL (T/S ratio ±SD)	1.72 (0.33)
***Psychological measures***	
Cook Medley Hostility Scale (± SD)	17.14 (7.47)
Marlowe-Crowne Social Desirability (± SD)	18.67 (5.38)
Beck Depression Inventory-II (± SD)	7.64 (8.21)
Beck Anxiety Inventory (± SD)	5.50 (6.21)

CRP = C-reactive protein, TL = telomere length.

*Metabolic syndrome: met at least 3 criteria: elevations in BP, waist circumference, glucose and triglyceride levels, and/or a reduction of high density lipoprotein levels (HDL)

#### Correlations among the psychological variables

Hostility, defensiveness, and symptoms of depression and anxiety were all significantly correlated with each other. See Table 2.

**Table 2:** Correlations among the psychological variables of interest.

Psychological variables	CMHo	MCSD	BAI
**CMHo**	**-**		
**MCSD**	-0.400**	-	
**BAI**	0.285**	-0.269**	-
**BDI-II**	0.308**	-0.274**	0.706**

**p<0.01;

CMHo:Cook-Medley Hostility Inventory; MCSD: Marlowe-Crowne Social Desirability Scale; BAI: Beck Anxiety Inventory; BDI-II: Beck Depression Inventory II

#### Univariate associations between TL and psychological variables

Greater defensiveness but lower hostility and anxiety were associated with shorter TL. No significant associations were found between depressive symptoms and TL. See Table 3.

**Table 3:** Univariate Pearson correlations between TL and psychological variables.

Psychological Variables	Telomere Length
**MCSD**	-0.330**
**CMHo**	0.323**
**BAI**	0.194*
**BDI-II**	0.034

*p<0.05,

**p<0.01;

CMHo: Cook-Medley Hostility Inventory; MCSD: Marlowe-Crowne Social Desirability Scale; BAI: Beck Anxiety Inventory; BDI-II: Beck Depression Inventory-II

#### Multivariate and independent associations of psychological variables with TL

Greater defensiveness and depressive symptoms were associated with significantly shorter TL, while greater hostility and anxiety symptoms were associated with longer TL. The psychological variables combined explained 19% of the variance over and above that explained by the covariates, and their associations with TL were independent of each other and the other covariates. While the psychological variables were significantly correlated with each other, no significant multicollinearity was found in the analysis (VIF: 1.264–1.908; Tolerance: 0.524–0.791). See Table 4 for the detailed results of the hierarchical linear regression model.

**Table 4:** Concurrent associations of psychological variables with TL.

**Block 1**	**β**	**t**	***p***	**Semi-partial r**
**Age**	-0.173	-1.945	0.054	-0.164
**Exercise or Not**	0.148	1.618	0.108	0.137
**CRP**	-0.103	-1.132	0.260	-0.096
**MAP**	-0.076	-0.855	0.394	-0.072
**#Alcoholic Beverages**	-0.108	-1.205	0.230	-0.102
F = (5,126) = 2.777, p = 0.020; R^2^ = 0.099; R^2^_adj_ = 0.064				
**Block 2**	**β**	**t**	***p***	**Semi-partial r**
**CMHo**	0.256	2.982	0.003	0.227
**MCSD**	-0.221	-2.525	0.013	-0.193
**BAI**	0.220	2.102	0.038	0.160
**BDI-II**	-0.213	-2.025	0.045	-0.154
F = (4,122) = 8.221, p<0.001R^2^ = 0.290; R^2^_adj_ = 0.20; R^2^_add_ = 0.19				

CRP: C-reactive protein; MAP: mean arterial pressure; CMHo: Cook-Medley Hostility Inventory; MCSD: Marlowe-Crowne Social Desirability Scale; BAI: Beck Anxiety Inventory; BDI-II: Beck Depression Inventory-II

#### Moderating effects of sex and age on the relation between TL and each psychological variable

Table 5 presents the detailed results of the four hierarchical regressions.

**Table 5:** Detailed summary of concurrent associations between individual psychological factors and TL.

**Block 1**	**β**	**t**	***p***	**Semi-partial r**
**Age**	-0.181	-2.005	0.047	-0.170
**Sex**	0.052	0.590	0.556	0.050
**Exercise**	0.150	-1.634	0.105	0.139
**CRP**	-0.109	-1.183	0.239	-0.100
**MAP**	-0.069	-0.768	0.444	-0.065
**#Alcoholic beverages**	-0.098	-1.078	0.283	-0.091
F _model_(6,125) = 2.361, *p*<0.05; R^2^_model_ = 0.102, R^2^_adj_ = 0.059				
**Hostility**				
**Block 2**	**β**	**t**	***p***	**Semi-partial r**
CMHo	0.362	4.500	<0.001	0.355
F_model_(1,124) = 20.249, *p*<0.001; R^2^_model_ = 0.228, R^2^_adj_ = 0.184				
**Social Desirability**				
**Block 2**	**β**	**t**	***p***	**Semi-partial r**
MCSD	-0.323	-3.993	<0.001	-0.320
F_model_(1,124) = 15.943, p<0.001; R^2^_model_ = 0.208, R^2^_adj_ = 0.159				
**Block 3**	**β**	**t**	***p***	**Semi-partial r**
Age*MCSD	0.179	2.198	0.030	0.173
F_model_(1,123) = 4.832, *p* = 0.030; R^2^_model_ = 0.234, R^2^_adj_ = 0.184				
**Symptoms of Depression**				
**Block 2**	**β**	**t**	***p***	**Semi-partial r**
BDI-II_log_	0.070	0.800	0.425	0.139
F _model_(1, 124) = 0.640, p = 0.425; R^2^_model_ = 0.106, R^2^_adj_ = 0.056				
**Symptoms of anxiety**				
**Block 2**	**β**	**t**	***p***	**Semi-partial r**
BAI_log_	0.223	2.623	0.010	0.217
F _model_ (1,124) = 6.878, *p* = 0.010; R^2^_model_ = 0.149, R^2^_adj_ = 0.101				

CRP: C-reactive protein; MAP: Mean Arterial Pressure

**Hostility and Anxiety:** The main effects of Hostility and Anxiety were significant, with both associated with significantly longer TL. Sex and/or age did not moderate these relations.

**Fig 1:**
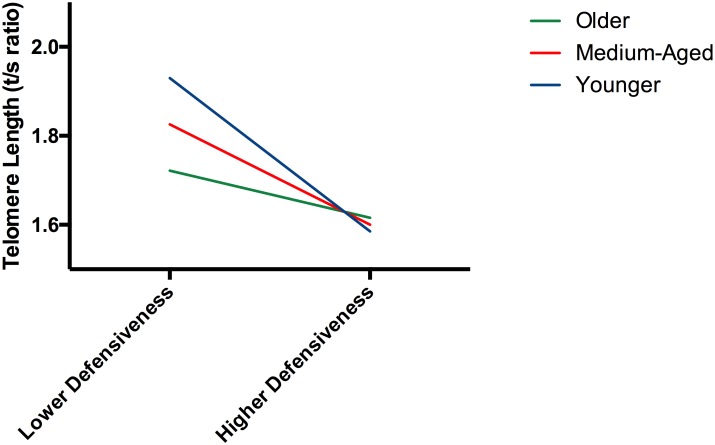
The relation between defensiveness and TL is moderated by age. The Age *by* Defensiveness interaction was significant. Greater defensiveness was associated with significantly shorter TL among young (b = -0.032, p = 0.001) and mid-aged individuals (b = -0.021, p<0.001) but not among older individuals (b = -0.001, p = 0.173), though the direction of effect was similar.

**Defensiveness trait:** The main effect of defensiveness was significant, with greater Defensiveness associated with shorter TL. The Age *by* Defensiveness interaction was also significant. Simple slope analyses indicated that greater defensiveness was associated with significantly shorter TL among young (b = -0.032, p = 0.001) and mid-aged individuals (b = -0.021, p<0.001) but not among older individuals (b = -0.001, p = 0.173) (Fig 1).

**Depression symptoms:** The main effect of depression was not significant. Sex and/or age did not moderate this relation.

## Post-hoc analyses

We examined whether the associations of distress variables with TL may reflect differential physiological responses to psychological stress. While TL tended to correlate with LF/HF-HRV (r = -0.163, p = 0.065) and SBP (r = -0.124, p = 0.072) reactivity, as well as with salivary cortisol (r = -0.160, p = 0.072), controlling for stress reactivity did not alter the results.

Findings for hostility and anxiety were unexpected. To increase our confidence in these results, we examined whether changes in these measures over the three-year period from Time 1 to Time 2 were similarly associated with TL at the follow-up assessment. Hierarchical regressions were performed separately for each psychological variable. Block 1 contained the covariates as per the main analyses, in addition to the Time 1 value for the psychological variable. The change score for the psychological variable (Time 2 –Time 1) was entered in Block 2. An increase in defensiveness over the 3 year period was associated with significantly shorter TL at Time 2 (β = -0.238, p = 0.009), whereas an increase in hostility over the same period was associated with longer TL (β = 0.234, p = 0.007). Changes in anxiety and depressive symptoms were not related to TL.

## Discussion

The aim of the present research was to examine the concurrent relations between psychological burden and TL, and to verify whether these associations were moderated by sex and/or age. The hypothesis that psychological distress variables would be associated with shorter telomere length was partly corroborated. More specifically, greater defensiveness was associated with shorter TL. On the other hand, greater hostility and anxiety were associated with longer, not shorter, telomeres. Depression showed inconsistent associations across the various analyses. Age moderated the associations of TL with defensiveness.

While some studies have found self-reported symptoms of depression to predict shorter telomere length [29, 86], results have been less consistent than with diagnosed mood disorders (see recent meta-analysis [87]). A novel finding in this study is that comorbid psychological states may influence the association of depression with TL. Indeed, in the current study, depressive symptoms predicted significantly shorter TL only when adjusting further for comorbid elevations in other psychological factors, particularly anxiety. This result did not appear to be a result of collinearity. It has been suggested that self-report questionnaires of depression may reflect acute emotional states that may have less bearing on biological processes involving TL in comparison to clinically diagnosed depressive disorders [25]. In the current sample, however, depressive symptoms appeared rather stable over the three-year period. Nonetheless, in individuals with other comorbid psychological difficulties, the impact of depressive symptoms may have been further augmented.

This is the first investigation to show that greater defensiveness is associated with shorter TL, particularly among younger individuals. Why this was not significant among our older participants is not clear, but may reflect certain resilience to the effects of defensiveness among this group, who were indeed rather healthy for their age. Alternatively, as individuals age, they become somewhat less dependent on or susceptible to the influence of social pressure for their wellbeing [88], which may reduce the impact of defensiveness on TL among older adults. Nonetheless, associations of defensiveness with higher arterial blood pressure, metabolic burden [54, 89] and now shorter TL, provide indications as to some of the mechanisms by which it may contribute to CVD development or prognosis.

Particularly surprising were our results relating to hostility and anxiety symptoms. The association of hostility with TL had only been examined once in the Whitehall II Cohort. It was found that among healthy older men, but not women, higher scores on the cynical subscale of the Cook-Medley Hostility Inventory were associated with significantly shorter TL [34, 90]. In contrast, we observed longer not shorter TL among more hostile men and women (whether using the full or abbreviated versions of the CMHo). Differential results may reflect the more heterogeneous nature of our sample compared to that of the Whitehall-II investigation. The latter was performed in white collar workers whereas our group had varied occupations. In terms of age, participants of the current study were generally younger and spanned a greater age range (Mean = 45 ± 11.6; Range 22–66 years versus Mean = 63 ± 5.6; Range 54–76 years). This reduced variability in their sample likely explains why they showed no association between TL and age as opposed to our study which showed the expected negative association (r = -0.187, p = 0.016). Alternatively, cultural differences in the meaning, expression, or understanding of hostility between our mostly Francophone sample and that of the English Whitehall cohort II may have contributed to contradictory results. However, several factors argue against this hypothesis. First, the Cook-Medley Hostility Scale shows similar psychometric properties in both samples. Moreover, we have previously shown the expected positive associations between hostility, metabolic abnormalities, and inflammatory activity [58, 91] in this same sample. Finally, the Francophone community within the greater Montreal region is rather heterogeneous and exposed to Anglophone media and culture. In sum, we have no reason to believe, at this time, that hostility means something different to our sample than it does to Americans or British individuals, nor that the hostility trait measure used was in any way inadequate for our population (see S1 Table for a comparison of psychometric properties of hostility questionnaires sampled from studies performed in different countries [34, 92–97]).

More anxious participants also showed longer TL in the current study. Boks and colleagues [98] similarly reported longer TL in military men with more self-reported PTSD symptoms six months after deployment in Afghanistan. In contrast, others have found null [24, 44] or negative associations between anxiety and TL among community samples or patients with anxiety disorders [99].

Given these unexpected results with anxiety and hostility, additional analyses were performed. To limit the possibility that the results reflected measurement error, TL was assayed a second time, in triplicate, with the same results. Post-hoc analyses were performed to examine whether increases in hostility and anxiety over a three year period were similarly associated with longer TL at follow-up, and at least for hostility, this was indeed the case, adding further credence to the results. As individual differences in leukocyte TL appear to be rather stable over time [100], it is possible that the more hostile or anxious participants in the current sample were, for some unknown reason, born with longer TL. This may have contributed to artificially elevated TL at our measurement point despite potentially greater telomeric loss over time. Alternatively, individuals with initially short leukocyte telomeres tend to show a slower rate of telomere shortening over time compared to those with longer telomeres [101, 102]. Telomerase protein activation and end-to-end recombination activities may contribute to telomere lengthening or maintenance in such cases [103]. While speculative at this stage, it is possible that compensatory increases in telomerase may have contributed to longer TL in more hostile or anxious individuals. This is consistent with findings by Epel and colleagues [104] who observed an increase in leukocyte telomerase activity following exposure to the Trier Social Stress task in postmenopausal women. Greater susceptibility and reactivity to stress among more anxious or hostile individuals [105, 106] may thus lead to over-expression of telomerase, with consequent elongation or at least greater preservation of TL in these individuals. As telomerase activity was not measured in this sample and TL was obtained only at one time point, we cannot verify these hypotheses.

It is difficult to reconcile why such compensatory mechanisms would be more greatly activated among hostile/anxious individuals, and not among those more depressed or showing greater defensiveness. This is all the more true given the high comorbidity among these various psychological factors. Behavioural and physiological differences may have contributed to differential profiles in TL. While our post-hoc analyses suggest that stress responses were not responsible for our findings, at least as measured concurrently, we have shown greater variability in interpersonal behaviours among more anxious versus depressed participants in this same sample [60]. While perhaps less predictable compared to depressed individuals, anxious individuals may be more adaptable in various life situations. This may translate into less adverse effects, at least on some biological processes in more anxious versus more depressed individuals. This is coherent with findings of more consistent associations of depression with CVD risk compared to anxiety [107, 108].

Alternatively, while longer TL is typically considered to be healthier, there is data to suggest that longer TL may in some cases be more pathogenic. Longer TL has been reported in individuals with left ventricular hypertrophy compared with control subjects [109, 110]. Longer TL in peripheral blood leukocytes has also been associated with a significantly increased risk of endometrial cancer [111]. Moreover, individuals with longer TL at diagnosis of either Parkinson's disease or Progressive Supranuclear Palsy respectively had an increased risk of developing dementia three years later [112]. Genetic factors may play a role in the relations observed. Indeed, greater hostility, anxiety, and TL in these individuals may reflect common genetic variance. For example, the *s* allele of the serotonin transporter (5-HTTLPR) gene has been associated with traits including anxiety and hostility in psychiatrically healthy populations [113, 114] and in a recent investigation, with TL [115].

Several limitations temper the conclusions that can be drawn from the current study. The sample size was relatively small, and moderator analyses may actually have over-fit the data. Analyses were cross-sectional, as such, it is impossible to infer causality or direction of effect. The healthy status of the sample, particularly in the older participants, may have limited the power to detect significant differences in TL or alternatively have led to the inclusion of highly resilient individuals not representative of the general population for this age group. On the other hand, the inclusion of relatively healthy individuals allows the evaluation of relations between variables of interest that are unconfounded by the effects of disease processes on these same variables. Moreover, the sample consisted of mostly French speaking Caucasians which may limit the generalizability of our study to other ethnicities [116, 117].

Conversely, results were particularly robust showing independent associations of the anxiety and personality traits with TL in both univariate and multivariate analyses that controlled for important socio-demographic, lifestyle, and physiological risk factors. To our knowledge, this is the first study to date that has shown the independence of associations from other psychological risk factors in a non-psychiatric population. Formal examination of whether age and/or sex moderated the relationships between psychological variables and TL were an added strength.

In conclusion, this study contributes to a growing body of evidence suggesting that personality traits and psychological symptoms influence TL. The mechanisms through which these psychological factors impact on TL remain to be elucidated. As observed in the current cross-sectional analyses, individual differences in concurrent health behaviours, cardiovascular or inflammatory processes, and acute stress responses do not appear be the main pathways of action. Further research is required to understand the underlying mechanisms involved in the relation between telomere length and psychological factors, as well as their prognostic significance.

## Supporting Information

S1 Table Psychometric properties of trait hostility questionnaires sampled across studies from different nations. ^*a*^*Psychological Profiles in the Prediction of Leukocyte Telomere Length in Healthy Individuals* (our manuscript)
(PDF)

## References

[1] Everson-Rose SA, Roetker NS, Lutsey PL, Kershaw KN, Longstreth WT Jr., Sacco RL, et al Chronic stress, depressive symptoms, anger, hostility, and risk of stroke and transient ischemic attack in the multi-ethnic study of atherosclerosis. Stroke. 2014;45(8):2318–23. 10.1161/STROKEAHA.114.004815 25013018 PMC4131200

[2] Pragodpol P, Ryan C. Critical review of factors predicting health-related quality of life in newly diagnosed coronary artery disease patients. The Journal of cardiovascular nursing. 2013;28(3):277–84. 10.1097/JCN.0b013e31824af56e 22495801

[3] Chida Y, Steptoe A. The association of anger and hostility with future coronary heart disease: a meta-analytic review of prospective evidence. Journal of the American College of Cardiology. 2009;53(11):936–46. 10.1016/j.jacc.2008.11.044 19281923

[4] Stafford M, Soljak M, Pledge V, Mindell J. Socio-economic differences in the health-related quality of life impact of cardiovascular conditions. European journal of public health. 2012;22(3):301–5. 10.1093/eurpub/ckr007 21398378 PMC3358629

[5] Spaderna H, Vogele C, Barten MJ, Smits JM, Bunyamin V, Weidner G. Physical activity and depression predict event-free survival in heart transplant candidates. Health psychology: official journal of the Division of Health Psychology, American Psychological Association. 2014;33(11):1328–36. 10.1037/hea0000033 24512323

[6] Mann SJ, James GD. Defensiveness and essential hypertension. Journal of psychosomatic research. 1998;45(2):139–48. 9753386 10.1016/s0022-3999(97)00293-6

[7] Khan FM, Kulaksizoglu B, Cilingiroglu M. Depression and coronary heart disease. Current atherosclerosis reports. 2010;12(2):105–9. 10.1007/s11883-010-0096-5 20425245

[8] Khayyam-Nekouei Z, Neshatdoost H, Yousefy A, Sadeghi M, Manshaee G. Psychological factors and coronary heart disease. ARYA atherosclerosis. 2013;9(1):102–11. 23690809 PMC3653260

[9] Rafanelli C, Gostoli S, Tully PJ, Roncuzzi R. Hostility and the clinical course of outpatients with congestive heart failure. Psychology & health. 2015:1–11. 10.1080/08870446.2015.1095299 26387801

[10] Compare A, Zarbo C, Manzoni GM, Castelnuovo G, Baldassari E, Bonardi A, et al Social support, depression, and heart disease: a ten year literature review. Frontiers in psychology. 2013;4:384 10.3389/fpsyg.2013.00384 23847561 PMC3696881

[11] Carty CL, Kooperberg C, Liu J, Herndon M, Assimes T, Hou L, et al Leukocyte Telomere Length and Risks of Incident Coronary Heart Disease and Mortality in a Racially Diverse Population of Postmenopausal Women. Arteriosclerosis, thrombosis, and vascular biology. 2015;35(10):2225–31. 10.1161/ATVBAHA.115.305838 26249011 PMC4713196

[12] Haycock PC, Heydon EE, Kaptoge S, Butterworth AS, Thompson A, Willeit P. Leucocyte telomere length and risk of cardiovascular disease: systematic review and meta-analysis. BMJ. 2014;349:g4227 10.1136/bmj.g4227 25006006 PMC4086028

[13] Blackburn EH. Telomere states and cell fates. Nature. 2000;408(6808):53–6. 10.1038/35040500 11081503

[14] Raschenberger J, Kollerits B, Titze S, Kottgen A, Barthlein B, Ekici AB, et al Association of relative telomere length with cardiovascular disease in a large chronic kidney disease cohort: the GCKD study. Atherosclerosis. 2015;242(2):529–34. 10.1016/j.atherosclerosis.2015.08.020 26302167

[15] Olovnikov AM. Telomeres, telomerase, and aging: origin of the theory. Experimental gerontology. 1996;31(4):443–8. 9415101 10.1016/0531-5565(96)00005-8

[16] Farzaneh-Far R, Cawthon RM, Na B, Browner WS, Schiller NB, Whooley MA. Prognostic value of leukocyte telomere length in patients with stable coronary artery disease: data from the Heart and Soul Study. Arteriosclerosis, thrombosis, and vascular biology. 2008;28(7):1379–84. 10.1161/ATVBAHA.108.167049 18467646 PMC2675880

[17] Willeit P, Willeit J, Brandstatter A, Ehrlenbach S, Mayr A, Gasperi A, et al Cellular ging reflected by leukocyte telomere length predicts advanced atherosclerosis and cardiovascular disease risk. Arteriosclerosis, thrombosis, and vascular biology. 2010;30(8):1649–56. 10.1161/ATVBAHA.110.205492 20508208

[18] Codd V, Nelson CP, Albrecht E, Mangino M, Deelen J, Buxton JL, et al Identification of seven loci affecting mean telomere length and their association with disease. Nature genetics. 2013;45(4):422–7, 7e1–2. 10.1038/ng.2528 23535734 PMC4006270

[19] Wu X, Amos CI, Zhu Y, Zhao H, Grossman BH, Shay JW, et al Telomere dysfunction: a potential cancer predisposition factor. Journal of the National Cancer Institute. 2003;95(16):1211–8. 12928346 10.1093/jnci/djg011

[20] Okereke OI, Prescott J, Wong JY, Han J, Rexrode KM, De Vivo I. High phobic anxiety is related to lower leukocyte telomere length in women. PloS one. 2012;7(7):e40516 10.1371/journal.pone.0040516 22808180 PMC3394740

[21] Ladwig KH, Brockhaus AC, Baumert J, Lukaschek K, Emeny RT, Kruse J, et al Posttraumatic stress disorder and not depression is associated with shorter leukocyte telomere length: findings from 3,000 participants in the population-based KORA F4 study. PloS one. 2013;8(7):e64762 10.1371/journal.pone.0064762 23843935 PMC3700974

[22] Wolkowitz OM, Mellon SH, Epel ES, Lin J, Dhabhar FS, Su Y, et al Leukocyte telomere length in major depression: correlations with chronicity, inflammation and oxidative stress—preliminary findings. PloS one. 2011;6(3):e17837 10.1371/journal.pone.0017837 21448457 PMC3063175

[23] Hartmann N, Boehner M, Groenen F, Kalb R. Telomere length of patients with major depression is shortened but independent from therapy and severity of the disease. Depression and anxiety. 2010;27(12):1111–6. 10.1002/da.20749 21053332

[24] Needham BL, Mezuk B, Bareis N, Lin J, Blackburn EH, Epel ES. Depression, anxiety and telomere length in young adults: evidence from the National Health and Nutrition Examination Survey. Molecular psychiatry. 2015;20(4):520–8. 10.1038/mp.2014.89 25178165 PMC4346549

[25] Wikgren M, Maripuu M, Karlsson T, Nordfjall K, Bergdahl J, Hultdin J, et al Short telomeres in depression and the general population are associated with a hypocortisolemic state. Biological psychiatry. 2012;71(4):294–300. 10.1016/j.biopsych.2011.09.015 22055018

[26] Needham BL, Adler N, Gregorich S, Rehkopf D, Lin J, Blackburn EH, et al Socioeconomic status, health behavior, and leukocyte telomere length in the National Health and Nutrition Examination Survey, 1999–2002. Social science & medicine. 2013;85:1–8. 23540359 10.1016/j.socscimed.2013.02.023 PMC3666871

[27] Simon NM, Smoller JW, McNamara KL, Maser RS, Zalta AK, Pollack MH, et al Telomere shortening and mood disorders: preliminary support for a chronic stress model of accelerated aging. Biological psychiatry. 2006;60(5):432–5. 10.1016/j.biopsych.2006.02.004 16581033

[28] Verhoeven JE, Revesz D, Epel ES, Lin J, Wolkowitz OM, Penninx BW. Major depressive disorder and accelerated cellular aging: results from a large psychiatric cohort study. Molecular psychiatry. 2014;19(8):895–901. 10.1038/mp.2013.151 24217256

[29] Huzen J, van der Harst P, de Boer RA, Lesman-Leegte I, Voors AA, van Gilst WH, et al Telomere length and psychological well-being in patients with chronic heart failure. Age and ageing. 2010;39(2):223–7. 10.1093/ageing/afp256 20085922

[30] Teyssier JR, Chauvet-Gelinier JC, Ragot S, Bonin B. Up-regulation of leucocytes genes implicated in telomere dysfunction and cellular senescence correlates with depression and anxiety severity scores. PloS one. 2012;7(11):e49677 10.1371/journal.pone.0049677 23185405 PMC3504145

[31] Hoen PW, de Jonge P, Na BY, Farzaneh-Far R, Epel E, Lin J, et al Depression and leukocyte telomere length in patients with coronary heart disease: data from the Heart and Soul Study. Psychosomatic medicine. 2011;73(7):541–7. 10.1097/PSY.0b013e31821b1f6e 21597035 PMC3161173

[32] Shaffer JA, Epel E, Kang MS, Ye S, Schwartz JE, Davidson KW, et al Depressive symptoms are not associated with leukocyte telomere length: findings from the Nova Scotia Health Survey (NSHS95), a population-based study. PloS one. 2012;7(10):e48318 10.1371/journal.pone.0048318 23133583 PMC3485011

[33] Hoen PW, Rosmalen JG, Schoevers RA, Huzen J, van der Harst P, de Jonge P. Association between anxiety but not depressive disorders and leukocyte telomere length after 2 years of follow-up in a population-based sample. Psychological medicine. 2013;43(4):689–97. 10.1017/S0033291712001766 22877856

[34] Brydon L, Lin J, Butcher L, Hamer M, Erusalimsky JD, Blackburn EH, et al Hostility and cellular aging in men from the Whitehall II cohort. Biological psychiatry. 2012;71(9):767–73. 10.1016/j.biopsych.2011.08.020 21974787 PMC3657139

[35] Starkweather AR, Sherwood P, Lyon DE, Bovbjerg DH, Broaddus WC, Elswick RK Jr., et al Depressive symptoms and cytokine levels in Serum and Tumor Tissue in patients with an Astrocytoma: a pilot study. BMC research notes. 2014;7:423 10.1186/1756-0500-7-423 24997057 PMC4118281

[36] Muezzinler A, Zaineddin AK, Brenner H. A systematic review of leukocyte telomere length and age in adults. Ageing research reviews. 2013;12(2):509–19. 10.1016/j.arr.2013.01.003 23333817

[37] Somers JM, Goldner EM, Waraich P, Hsu L. Prevalence and incidence studies of anxiety disorders: a systematic review of the literature. Canadian journal of psychiatry Revue canadienne de psychiatrie. 2006;51(2):100–13. 16989109 10.1177/070674370605100206

[38] Gardner M, Bann D, Wiley L, Cooper R, Hardy R, Nitsch D, et al Gender and telomere length: systematic review and meta-analysis. Experimental gerontology. 2014;51:15–27. 10.1016/j.exger.2013.12.004 24365661 PMC4523138

[39] Okuda K, Bardeguez A, Gardner JP, Rodriguez P, Ganesh V, Kimura M, et al Telomere length in the newborn. Pediatric research. 2002;52(3):377–81. 10.1203/00006450-200209000-00012 12193671

[40] Hunt SC, Chen W, Gardner JP, Kimura M, Srinivasan SR, Eckfeldt JH, et al Leukocyte telomeres are longer in African Americans than in whites: the National Heart, Lung, and Blood Institute Family Heart Study and the Bogalusa Heart Study. Aging cell. 2008;7(4):451–8. 10.1111/j.1474-9726.2008.00397.x 18462274 PMC2810865

[41] Piccinelli M, Wilkinson G. Gender differences in depression. Critical review. The British journal of psychiatry: the journal of mental science. 2000;177:486–92. 11102321 10.1192/bjp.177.6.486

[42] Eaton WW, Kramer M, Anthony JC, Dryman A, Shapiro S, Locke BZ. The incidence of specific DIS/DSM-III mental disorders: data from the NIMH Epidemiologic Catchment Area Program. Acta psychiatrica Scandinavica. 1989;79(2):163–78. 2784251 10.1111/j.1600-0447.1989.tb08584.x

[43] Weisberg YJ, Deyoung CG, Hirsh JB. Gender Differences in Personality across the Ten Aspects of the Big Five. Frontiers in psychology. 2011;2:178 10.3389/fpsyg.2011.00178 21866227 PMC3149680

[44] Kananen L, Surakka I, Pirkola S, Suvisaari J, Lonnqvist J, Peltonen L, et al Childhood adversities are associated with shorter telomere length at adult age both in individuals with an anxiety disorder and controls. PloS one. 2010;5(5):e10826 10.1371/journal.pone.0010826 20520834 PMC2876034

[45] Crowne DP, Marlowe D. A new scale of social desirability independent of psychopathology. J Consult Psychol. 1960;24:349–54. 13813058 10.1037/h0047358

[46] Paulhus DL. Two-component models of socially desirable responding. Journal of personality and social psychology. 1984;46(3):598.

[47] Helmers KF, Krantz DS, Merz CN, Klein J, Kop WJ, Gottdiener JS, et al Defensive hostility: relationship to multiple markers of cardiac ischemia in patients with coronary disease. Health psychology: official journal of the Division of Health Psychology, American Psychological Association. 1995;14(3):202–9. 10.1037//0278-6133.14.3.202 7641660

[48] Jorgensen RS, Frankowski JJ, Lantinga LJ, Phadke K, Sprafkin RP, Abdul-Karim KW. Defensive hostility and coronary heart disease: a preliminary investigation of male veterans. Psychosomatic medicine. 2001;63(3):463–9. 11382274 10.1097/00006842-200105000-00016

[49] Denollet J. Negative affectivity and repressive coping: pervasive influence on self-reported mood, health, and coronary-prone behavior. Psychosomatic medicine. 1991;53(5):538–56. 1758940 10.1097/00006842-199109000-00005

[50] Rutledge T, Linden W. Defensiveness and 3-year blood pressure levels among young adults: the mediating effect of stress-reactivity. Annals of behavioral medicine: a publication of the Society of Behavioral Medicine. 2003;25(1):34–40. 12581934 10.1207/S15324796ABM2501_05

[51] Nyklíček I, Vingerhoets A, Van Heck G, Van Limpt M. Defensive coping in relation to casual blood pressure and self-reported daily hassles and life events. Journal of behavioral medicine. 1998;21(2):145–61. 9591167 10.1023/a:1018775807593

[52] Rutledge T, Hogan BE. A quantitative review of prospective evidence linking psychological factors with hypertension development. Psychosomatic medicine. 2002;64(5):758–66. 12271106 10.1097/01.psy.0000031578.42041.1c

[53] Levesque K, Moskowitz DS, Tardif JC, Dupuis G, D'Antono B. Physiological stress responses in defensive individuals: age and sex matter. Psychophysiology. 2010;47(2):332–41. 10.1111/j.1469-8986.2009.00943.x 20070571

[54] Lévesque K, Bureau S, Moskowitz DS, Tardif JC, Lavoie J, Dupuis G, et al Defensiveness and metabolic syndrome: impact of sex and age. Biol Psychol. 2009;80(3):354–60. 10.1016/j.biopsycho.2008.12.003 19150480

[55] Gentile C, Dragomir AI, Solomon C, Nigam A, D'Antono B. Sex differences in the prediction of metabolic burden from physiological responses to stress. Annals of behavioral medicine: a publication of the Society of Behavioral Medicine. 2015;49(1):112–27. 25228454 10.1007/s12160-014-9639-2

[56] Gordon JL, Ditto B, D'Antono B. Cognitive depressive symptoms associated with delayed heart rate recovery following interpersonal stress in healthy men and women. Psychophysiology. 2012;49(8):1082–9. 10.1111/j.1469-8986.2012.01397.x 22725718

[57] Juster RP, Moskowitz DS, Lavoie J, D'Antono B. Sex-specific interaction effects of age, occupational status, and workplace stress on psychiatric symptoms and allostatic load among healthy Montreal workers. Stress. 2013;16(6):616–29. 10.3109/10253890.2013.835395 23952366

[58] Boisclair Demarble J, Moskowitz DS, Tardif JC, D'Antono B. The relation between hostility and concurrent levels of inflammation is sex, age, and measure dependent. Journal of psychosomatic research. 2014;76(5):384–93. 10.1016/j.jpsychores.2014.02.010 24745780

[59] Dragomir AI, Gentile C, Nolan RP, D'Antono B. Three-year stability of cardiovascular and autonomic nervous system responses to psychological stress. Psychophysiology. 2014;51(9):921–31. 10.1111/psyp.12231 24853995

[60] Rappaport LM, Moskowitz DS, D'Antono B. Naturalistic interpersonal behavior patterns differentiate depression and anxiety symptoms in the community. Journal of counseling psychology. 2014;61(2):253–63. 10.1037/a0035625 24660689

[61] Beck AT, Epstein N, Brown G, Steer RA. An inventory for measuring clinical anxiety: psychometric properties. J Consult Clin Psychol. 1988;56(6):893–7. 3204199 10.1037//0022-006x.56.6.893

[62] Beck AT, Steer RA, Brown GK. Beck depression inventory-II. San Antonio, TX: Psychological Corporation 1996:b9.

[63] Arnau RC, Meagher MW, Norris MP, Bramson R. Psychometric evaluation of the Beck Depression Inventory-II with primary care medical patients. Health Psychology. 2001;20(2):112 11315728 10.1037//0278-6133.20.2.112

[64] Cook WW, Medley DM. Proposed hostility and pharisaic-virtue scales for the MMPI. Journal of Applied Psychology. 1954;38(6):414.

[65] Smith TW, Frohm KD. What's so unhealthy about hostility? Construct validity and psychosocial correlates of the Cook and Medley Ho scale. Health Psychol. 1985;4(6):503–20. 3830702 10.1037//0278-6133.4.6.503

[66] Barefoot JC, Dahlstrom WG, Williams RB Jr. Hostility, CHD incidence, and total mortality: a 25-year follow-up study of 255 physicians. Psychosomatic medicine. 1983;45(1):59–63. 6844529 10.1097/00006842-198303000-00008

[67] Crowne DP, Marlowe D. A new scale of social desirability independent of psychopathology. Journal of consulting psychology. 1960;24(4):349. 13813058 10.1037/h0047358

[68] Crino MD, Svoboda M, Rubenfeld S, White MC. Data on the Marlowe-Crowne and Edwards social desirability scales. Psychological Reports. 1983;53(3):963–8.

[69] Cawthon RM. Telomere measurement by quantitative PCR. Nucleic acids research. 2002;30(10):e47 12000852 10.1093/nar/30.10.e47 PMC115301

[70] Mollica L, Fleury I, Belisle C, Provost S, Roy DC, Busque L. No association between telomere length and blood cell counts in elderly individuals. J Gerontol A Biol Sci Med Sci. 2009;64(9):965–7. 10.1093/gerona/glp065 19435952

[71] Llabre MM, Spitzer SB, Saab PG, Ironson GH, Schneiderman N. The reliability and specificity of delta versus residualized change as measures of cardiovascular reactivity to behavioral challenges. Psychophysiology. 1991;28(6):701–11. 1816598 10.1111/j.1469-8986.1991.tb01017.x

[72] Ludlow AT, Ludlow LW, Roth SM. Do telomeres adapt to physiological stress? Exploring the effect of exercise on telomere length and telomere-related proteins. BioMed research international. 2013;2013. 10.1155/2013/601368 PMC3884693 24455708

[73] Soares-Miranda L, Imamura F, Siscovick D, Jenny NS, Fitzpatrick AL, Mozaffarian D. Physical Activity, Physical Fitness, and Leukocyte Telomere Length: The Cardiovascular Health Study. Medicine and science in sports and exercise. 2015;47(12):2525–34. 10.1249/MSS.0000000000000720 26083773 PMC4648672

[74] Shin C, Baik I. Associations Between Alcohol Consumption and Leukocyte Telomere Length Modified by a Common Polymorphism of ALDH2. Alcoholism: Clinical and Experimental Research. 2016;40(4):765–71. 10.1111/acer.13005 26972231

[75] Pavanello S, Hoxha M, Dioni L, Bertazzi PA, Snenghi R, Nalesso A, et al Shortened telomeres in individuals with abuse in alcohol consumption. International Journal of Cancer. 2011;129(4):983–92. 10.1002/ijc.25999 21351086 PMC3125427

[76] Prasad K. C-reactive protein increases oxygen radical generation by neutrophils. Journal of cardiovascular pharmacology and therapeutics. 2004;9(3):203–9. 15378141 10.1177/107424840400900308

[77] von Zglinicki T. Oxidative stress shortens telomeres. Trends in biochemical sciences. 2002;27(7):339–44. 12114022 10.1016/s0968-0004(02)02110-2

[78] Pedroso DCC, Miranda-Furtado CL, Kogure GS, Meola J, Okuka M, Silva C, et al Inflammatory biomarkers and telomere length in women with polycystic ovary syndrome. Fertility and sterility. 2015;103(2):542–7. e2 10.1016/j.fertnstert.2014.10.035 25467041

[79] Rode L, Nordestgaard BG, Weischer M, Bojesen SE. Increased body mass index, elevated C-reactive protein, and short telomere length. The Journal of Clinical Endocrinology & Metabolism. 2014;99(9):E1671–E5. 24762112 10.1210/jc.2014-1161

[80] Smith TW, Allred KD. Blood-pressure responses during social interaction in high-and low-cynically hostile males. Journal of Behavioral Medicine. 1989;12(2):135–43. 2760919 10.1007/BF00846547

[81] Jorgensen RS, Frankowski JJ, Lantinga LJ, Phadke K, Sprafkin RP, Abdul-Karim KW. Defensive hostility and coronary heart disease: a preliminary investigation of male veterans. Psychosomatic Medicine. 2001;63(3):463–9. 11382274 10.1097/00006842-200105000-00016

[82] Markovitz JH, Matthews KA, Wing RR, Kuller LH, Meilahn EN. Psychological, biological and health behavior predictors of blood pressure changes in middle-aged women. Journal of Hypertension. 1991;9(5):399–406. 1649859 10.1097/00004872-199105000-00003

[83] Koh DJ, Kim NY, Kim YW. Predictors of Depressive Mood in Patients With Isolated Cerebellar Stroke: A Retrospective Study. Annals of Rehabilitation Medicine. 2016;40(3):412–9. 10.5535/arm.2016.40.3.412 27446777 PMC4951359

[84] Kim J-H, Kim HK, Ko J-H, Bang H, Lee D-C. The relationship between leukocyte mitochondrial DNA copy number and telomere length in community-dwelling elderly women. PLoS One. 2013;8(6):e67227 10.1371/journal.pone.0067227 23785520 PMC3681770

[85] Preacher KJ, Hayes AF. SPSS and SAS procedures for estimating indirect effects in simple mediation models. Behavior research methods, instruments, & computers: a journal of the Psychonomic Society, Inc. 2004;36(4):717–31. 10.3758/bf03206553 15641418

[86] Lin J, Blalock JA, Chen M, Ye Y, Gu J, Cohen L, et al Depressive symptoms and short telomere length are associated with increased mortality in bladder cancer patients. Cancer epidemiology, biomarkers & prevention: a publication of the American Association for Cancer Research, cosponsored by the American Society of Preventive Oncology. 2015;24(2):336–43. 10.1158/1055-9965.EPI-14-0992 PMC4332382 25416716

[87] Kinser PA, Lyon DE. Major depressive disorder and measures of cellular aging: an integrative review. Nursing research and practice. 2013;2013:469070 10.1155/2013/469070 23691300 PMC3649747

[88] Pasupathi M. Age differences in response to conformity pressure for emotional and nonemotional material. Psychology and aging. 1999;14(1):170–4. 10224640 10.1037//0882-7974.14.1.170

[89] Jorgensen RS, Johnson BT, Kolodziej ME, Schreer GE. Elevated blood pressure and personality: a meta-analytic review. Psychological bulletin. 1996;120(2):293–320. 8831299 10.1037/0033-2909.120.2.293

[90] Zalli A, Carvalho LA, Lin J, Hamer M, Erusalimsky JD, Blackburn EH, et al Shorter telomeres with high telomerase activity are associated with raised allostatic load and impoverished psychosocial resources. Proceedings of the National Academy of Sciences of the United States of America. 2014;111(12):4519–24. 10.1073/pnas.1322145111 24616496 PMC3970484

[91] D'Antono B, Moskowitz DS, Nigam A. The metabolic costs of hostility in healthy adult men and women: cross-sectional and prospective analyses. J Psychosom Res. 2013;75(3):262–9. 10.1016/j.jpsychores.2013.05.010 23972416

[92] Everson-Rose SA, Lewis TT, Karavolos K, Matthews KA, Sutton-Tyrrell K, Powell LH. Cynical hostility and carotid atherosclerosis in African American and white women: the Study of Women's Health Across the Nation (SWAN) Heart Study. American Heart Journal. 2006;152(5):982 e7- e13. 10.1016/j.ahj.2006.08.010 17070176

[93] Mwendwa DT, Ali MK, Sims RC, Madhere S, Levy S-A, Callender CO, et al Psychometric properties of the Cook Medley Hostility Scale and its association with inflammatory markers in African Americans. Psychology, health & medicine. 2013;18(4):431–44. 10.1080/13548506.2012.736623 23116190

[94] Lemogne C, Schuster J-P, Levenstein S, Melchior M, Nabi H, Ducimetière P, et al Hostility and the risk of peptic ulcer in the GAZEL cohort. Health Psychology. 2015;34(2):181 10.1037/hea0000129 25110845

[95] Sykes D, Arveiler D, Salters C, Ferrieres J, McCrum E, Amouyel P, et al Psychosocial risk factors for heart disease in France and Northern Ireland: the Prospective Epidemiological Study of Myocardial Infarction (PRIME). International journal of epidemiology. 2002;31(6):1227–34. 12540727 10.1093/ije/31.6.1227

[96] Bermúdez J, Sánchez-Elvira A, Fernández E, editors. Contenido del Inventario de Hostilidad de Cook y Medley (ICM): implicaciones pronocoronarias. Comunicación presentada al IV Congreso de Evaluación Psicológica, Santiago de Compostela; 1994.

[97] Miller SB, Dolgoy L, Friese M, Sita A. Parental history of hypertension and hostility moderate cardiovascular responses to interpersonal conflict. International Journal of Psychophysiology. 1998;28(2):193–206. 9545656 10.1016/s0167-8760(97)00096-2

[98] Boks MP, van Mierlo HC, Rutten BP, Radstake TR, De Witte L, Geuze E, et al Longitudinal changes of telomere length and epigenetic age related to traumatic stress and post-traumatic stress disorder. Psychoneuroendocrinology. 2015;51:506–12. 10.1016/j.psyneuen.2014.07.011 25129579

[99] Verhoeven JE, Revesz D, van Oppen P, Epel ES, Wolkowitz OM, Penninx BW. Anxiety disorders and accelerated cellular ageing. The British journal of psychiatry: the journal of mental science. 2015;206(5):371–8. 25657360 10.1192/bjp.bp.114.151027

[100] Shalev I, Moffitt TE, Braithwaite AW, Danese A, Fleming NI, Goldman-Mellor S, et al Internalizing disorders and leukocyte telomere erosion: a prospective study of depression, generalized anxiety disorder and post-traumatic stress disorder. Molecular psychiatry. 2014;19(11):1163–70. 10.1038/mp.2013.183 24419039 PMC4098012

[101] Nordfjall K, Svenson U, Norrback KF, Adolfsson R, Lenner P, Roos G. The individual blood cell telomere attrition rate is telomere length dependent. PLoS Genet. 2009;5(2):e1000375 10.1371/journal.pgen.1000375 19214207 PMC2633043

[102] Epel ES, Merkin SS, Cawthon R, Blackburn EH, Adler NE, Pletcher MJ, et al The rate of leukocyte telomere shortening predicts mortality from cardiovascular disease in elderly men. Aging (Albany NY). 2009;1(1):81–8. 10.18632/aging.100007 PMC2830080 20195384

[103] Blackburn EH, Collins K. Telomerase: an RNP enzyme synthesizes DNA. Cold Spring Harbor perspectives in biology. 2011;3(5). 10.1101/cshperspect.a003558 PMC3101848 20660025

[104] Epel ES, Lin J, Dhabhar FS, Wolkowitz OM, Puterman E, Karan L, et al Dynamics of telomerase activity in response to acute psychological stress. Brain, behavior, and immunity. 2010;24(4):531–9. 10.1016/j.bbi.2009.11.018 20018236 PMC2856774

[105] Robles TF, Carroll JE. Restorative biological processes and health. Social and personality psychology compass. 2011;5(8):518–37. 10.1111/j.1751-9004.2011.00368.x 21927619 PMC3171702

[106] Chida Y, Hamer M. Chronic psychosocial factors and acute physiological responses to laboratory-induced stress in healthy populations: a quantitative review of 30 years of investigations. Psychol Bull. 2008;134(6):829–85. 10.1037/a0013342 18954159

[107] Skilton MR, Moulin P, Terra JL, Bonnet F. Associations between anxiety, depression, and the metabolic syndrome. Biological psychiatry. 2007;62(11):1251–7. 10.1016/j.biopsych.2007.01.012 17553465

[108] Frasure-Smith N, Lesperance F. Recent evidence linking coronary heart disease and depression. Canadian journal of psychiatry Revue canadienne de psychiatrie. 2006;51(12):730–7. 17168247 10.1177/070674370605101202

[109] Vasan RS, Demissie S, Kimura M, Cupples LA, White C, Gardner JP, et al Association of leukocyte telomere length with echocardiographic left ventricular mass: the Framingham heart study. Circulation. 2009;120(13):1195–202. 10.1161/CIRCULATIONAHA.109.853895 19752323 PMC2857693

[110] Kuznetsova T, Codd V, Brouilette S, Thijs L, Gonzalez A, Jin Y, et al Association between left ventricular mass and telomere length in a population study. American journal of epidemiology. 2010;172(4):440–50. 10.1093/aje/kwq142 20660518

[111] Sun Y, Zhang L, Zhao L, Wu X, Gu J. Association of leukocyte telomere length in peripheral blood leukocytes with endometrial cancer risk in Caucasian Americans. Carcinogenesis. 2015;36(11):1327–32. 10.1093/carcin/bgv133 26385889

[112] Degerman S, Domellof M, Landfors M, Linder J, Lundin M, Haraldsson S, et al Long leukocyte telomere length at diagnosis is a risk factor for dementia progression in idiopathic parkinsonism. PloS one. 2014;9(12):e113387 10.1371/journal.pone.0113387 25501556 PMC4264694

[113] Gonda X, Fountoulakis KN, Juhasz G, Rihmer Z, Lazary J, Laszik A, et al Association of the s allele of the 5-HTTLPR with neuroticism-related traits and temperaments in a psychiatrically healthy population. Eur Arch Psychiatry Clin Neurosci. 2009;259(2):106–13. 10.1007/s00406-008-0842-7 18806915

[114] Lesch KP, Bengel D, Heils A, Sabol SZ, Greenberg BD, Petri S, et al Association of anxiety-related traits with a polymorphism in the serotonin transporter gene regulatory region. Science. 1996;274(5292):1527–31. 8929413 10.1126/science.274.5292.1527

[115] Li P, Liu T, Liu J, Zhang Q, Lou F, Kong F, et al Promoter polymorphism in the serotonin transporter (5-HTT) gene is significantly associated with leukocyte telomere length in Han Chinese. PloS one. 2014;9(4):e94442 10.1371/journal.pone.0094442 24710073 PMC3978058

[116] Geronimus AT, Pearson JA, Linnenbringer E, Schulz AJ, Reyes AG, Epel ES, et al Race-Ethnicity, Poverty, Urban Stressors, and Telomere Length in a Detroit Community-based Sample. Journal of health and social behavior. 2015;56(2):199–224. 10.1177/0022146515582100 25930147 PMC4621968

[117] Lynch SM, Peek MK, Mitra N, Ravichandran K, Branas C, Spangler E, et al Race, Ethnicity, Psychosocial Factors, and Telomere Length in a Multicenter Setting. PloS one. 2016;11(1):e0146723 10.1371/journal.pone.0146723 26752285 PMC4709232

