# Supplementary material for: Psychological Profiles in the Prediction of Leukocyte Telomere Length in Healthy Individuals
Source: PLoS One. 2016 Oct 27;11(10):e0165482. doi: 10.1371/journal.pone.0165482 (PMC5082938; doi:10.1371/journal.pone.0165482)
Supplement: S1 Table — aPsychological Profiles in the Prediction of Leukocyte Telomere Length in Healthy Individuals (our manuscript) (PDF) [file pone.0165482.s001.pdf]

S1Table. **Psychometric properties of trait hostility questionnaires sampled across studies from different nations**

Country-Culture	Questionnaire	Ranges/Means (SD)	Test Retest Reliability (r)	Internal Consistency (Cronbach's α)
**1. Montreal, Canada[a]**	Cook-Medley Hostility Scale (50 items)	2-39 **Mean:** 17.14 (7.47)	0.84 (3 years)	0.83
**2. United States (93)**	Cook-Medley Hostility Cynical subscale (13 items)	**Range** *NA* **Mean:** 3.57 (2.9)	0.88 (3 years)	0.79
**3. United States (94)**	Cook-Medley Hostility Scale (50 items)	1-49 **Mean**: 23.27 (7.82)	0.84 (4 year test-retest)	0.86
**4. United Kingdom (34)**	Cook-Medley Hostility Cynical subscale (10 items)	0-10 **Mean** : 2.55 (2.39)	*NA*	0.77
**5. France (95)**	Buss Durkey Hostility Inventory (63 items)	0-63 **Mean** : 25.8 (9.9)	0.87 (3 months test- retest)	0.87
**6. France and Northern Ireland (Belfast, Strasbourg, Toulouse, Lille) (96)**	Cook-Medley Hostility Cynical Distrust Scale (7 items)	0-7 **Means :** Belfast : 2.11 (1.84) Strasbourg : 3.77 (2.17) Toulouse :3.29 (2.18) Lille : 3.77 (2.14)	*NA*	0.75
**7. Spain (97)**	Cook-Medley Hostility Scale *Spanish Version* (50 items)	0-50 **Mean** : *NA*	0.75	0.80
**8. Montreal, Canada (54)**	Cook-Medley Hostility Scale (50 items)	(Time 1) 3-36 **Mean** : 18.47 (7.42)	0.84 (3 years)	0.83
**9. Montreal, Canada (98)**	Cook-Medley Hostility Scale (50 items)	*NA*	0.77	0.80

[a]*Psychological Profiles in the Prediction of Leukocyte Telomere Length in Healthy Individuals* (our manuscript)

Social desirability is often used as a covariate when examining other psychological responses or traits. Some may ask why we did not control for social desirability in analyses involving hostility and the affect items.

Response: It is true that the Marlowe-Crowne Social Desirability questionnaire has frequently been used as a covariate when examining other psychological responses or traits, in order to control for response biases, or the tendency to fake good. However, decades of research exist in which social desirability was examined as an important psychological trait and risk factor for cardiovascular disease in its own right: Defensiveness, measured most frequently using the Marlowe-Crowne, refers among other things, to a personality trait characterized by the avoidance, denial or repression of information (e.g., negative affects, physical symptoms, poor performance) perceived as threatening to the individual. Individuals with this trait have a tendency to behave in a socially desirable manner such as conforming to the opinion of others or lying about inappropriate behaviors and suboptimal performance in order to secure social bonds and/or to protect a vulnerable self-esteem(45, 46). Individuals who are more defensive are at greater risk for cardiovascular-related morbidity (47, 48) and mortality (49) and show a poorer prognosis following pharmacological treatment in hypertensive individuals (6, 50). It has been associated with elevations in blood pressure and hypertension in meta-analyses (90) and later cross-sectional and prospective studies (6, 50-52).In the current sample, defensiveness was associated with a worse metabolic profil (54).

Moreover, results for hostility and anxiety continued to show a significant positive relation with TL in analyses in which all psychological variables (including defensiveness) was entered. See Table 2 of the main manuscript.

For these reasons and because suddenly using a main predictor as a covariate would be confusing and redundant with Table 2, analyses were not altered in main or posthoc analyses.

While stress reactivity was related to TL and could have been examined as moderators, this was not examined in the current manuscript. It was not the objective of our study in an already lengthy paper and would in fact merit its own paper.
